# Retraction: Pre-infection 25-hydroxyvitamin D3 levels and association with severity of COVID-19 illness

**DOI:** 10.1371/journal.pone.0331693

**Published:** 2025-09-08

**Authors:** The PLOS One Editors

Following the publication of this article [1], concerns were raised about the validity of the reported methods and reliability of the conclusions drawn.

The article was reassessed by an independent member of the *PLOS One* Editorial Board after concerns were raised about the results presented in the article, who advised that the analyses performed were inadequate to test the association between 25-hydroxyvitamin D3 levels at the time of infection and severity of COVID-19 illness.

Specifically,

- The inclusion of only patients for whom a vitamin D determination had been performed before COVID-19 infection introduces bias.
- The potential medical reasons for a vitamin D evaluation prior to infection and the potential corrective therapy that may have been undertaken introduce major confounding factors that have not been adequately accounted for. This bias is further exacerbated because the vitamin D measurements used in the analyses were collected between 14–730 days before COVID-19 infection, without consideration of the potential changes in vitamin D levels — other than seasonality — in between the time of determination and the time of infection.

Consequently, the Editorial Board member concluded that the experimental design of the study has a fatal flaw which prevents testing of the hypothesis and which calls into question the reliability of the reported conclusions.

The authors responded to the concerns raised by the Editorial Board member, but the response did not resolve the concerns underlying the retraction decision.

In light of the above concerns pertaining to the reported experimental design and conclusions, the *PLOS One* Editors retract this article [1]. We regret that the issues were not identified prior to the article’s publication.

AAD, AD, YN, OY, YS, ESegal, LF, MM, NE, DR, MG, AB, EK, ME, and ESela did not agree with the retraction. NM, ML, and MB either did not respond directly or could not be reached.
